# Primary Care Psychiatry eConsults at a Rural Academic Medical Center: Descriptive Analysis

**DOI:** 10.2196/24650

**Published:** 2021-09-01

**Authors:** Jade Avery, Dennis Dwan, Gillian Sowden, Matthew Duncan

**Affiliations:** 1 Department of Psychiatry New York Presbyterian Columbia University New York City, NY United States; 2 Carney Hospital Dorchester, MA United States; 3 Department of Psychiatry Dartmouth-Hitchock Medical Center Lebanon, NH United States

**Keywords:** electronic consultation, psychiatry, eConsult, telehealth, telemedicine, rural

## Abstract

**Background:**

Primary care providers serve a crucial role in addressing the mental health needs of many patients. However, there are times when input from a psychiatric specialist may be helpful in supporting the mental health care provided in primary care. Psychiatry eConsults can serve as a valuable tool in providing specialist advice for primary care physicians when direct referral to specialty care is not readily available.

**Objective:**

The goal of this study is to evaluate the content and implementation of psychiatric eConsults by primary care providers in a rural academic medical center.

**Methods:**

This is a retrospective review of 343 eConsults placed between May 2016 and February 2019 by primary care providers at a single academic medical center. The content of eConsult requests, including patient diagnosis, consult question type, specialist recommendations, patient demographics, the distance of patient and primary care providers from the consulting provider, rate of implementation of the recommendation, and response time, were analyzed.

**Results:**

The most common diagnoses associated with eConsults were depression (162/450, 36%) and anxiety (118/450, 26%). The most commonly asked eConsult question was regarding medication management, including medication choice, side effects, interactions, and medication taper (288/343, 84%). More than one recommendation was included in 76% (259/343) of eConsults, and at least one recommendation was implemented by the primary care provider in 94% (282/300) of eConsults. The average time to respond to an eConsult was 26 hours.

**Conclusions:**

This study demonstrates that psychiatry eConsults can be conducted in a timely manner and that primary care providers implement the recommendations at a high rate.

## Introduction

Mental illness is especially prevalent, with nearly 1 in 5 adults meeting criteria for a psychiatric disorder [1]. Untreated mental illness has been associated with decreased functionality, reduced quality of life, heightened physical health complications, and premature mortality [2]. Although the demand for mental health treatment has been increasing, studies have demonstrated a shortage of mental health care clinicians, limited access to mental health care [3], and extensive wait times [4].

Patients in rural settings (in particular) face additional challenges in accessing mental health services. Rural populations are also faced with geographic isolation and greater stigmatization of mental health conditions [5]. Residents of small towns that are geographically far from larger metropolitan areas report significantly less treatment of mental health conditions than residents of cities, suburban areas, or rural areas adjacent to larger metropolitan areas [3]. Although primary care providers (PCPs) serve a crucial role in addressing the mental health needs of patients and providing access to care [6], referrals to specialists may be helpful in the treatment of many psychiatric conditions. Increasing rates of specialist referrals and a shortage of specialist availability represent a growing need for timely specialist advice and increased collaboration between PCPs and psychiatrists to reduce gaps in care.

The opportunity for collaboration between a PCP and psychiatrist can be achieved through eConsults [7]. eConsults are provider-to-provider communications within a shared electronic medical record or web-based platform. The use of eConsults has steadily expanded over the years, with eConsults serving as an opportunity to improve health care quality and reduce specialty care costs [7-10]. Although the use of eConsults has been established in several specialties—included but not limited to dermatology, cardiology, and nephrology—its use within the field of psychiatry has recently increased and may prove beneficial [11-13]. With extensive wait times for mental health prescribers in many areas, especially among rural populations, these consultations provide access to psychiatric specialists, may reduce delay in starting psychiatric treatment, and reach patients who may otherwise go without psychiatric care [7,14]. Although there are possible advantages for the use of psychiatric eConsults, little research has been conducted on the model of a psychiatric eConsult system in an academic rural health care setting.

The aim of this study is to explore the patterns, content, and provider implementation of recommendations of psychiatric eConsults in a large academic health care system. By conducting a descriptive analysis of 343 psychiatric eConsults, we outlined the practice of psychiatric eConsults, with the goal of portraying the acceptability and efficiency of this practice. We hope that our findings will add to the growing literature on e-consultation and show that a modest amount of specialist time can support PCPs in their delivery of mental health care for patients seeking help in primary care.

## Methods

### Implementation, Execution, and Structure of a Psychiatric Electronic Consultation Service Within a Large Academic System

Dartmouth-Hitchcock Health is a rural academic medical center with five primary care practice sites spread across New Hampshire. PCPs include attending physicians, resident physicians, physician assistants, and nurse practitioners. All clinics use Epic Systems software as a shared electronic health record (EHR). General electronic consultations were first launched at Dartmouth-Hitchcock Health in 2014 as part of the Association of American Medical Colleges (AAMC) Project CORE (Coordinating Optimal Referral Experiences: Implementing eConsults and Enhanced Referrals). The Department of Psychiatry joined the eConsult program at Dartmouth-Hitchcock Health in 2016. By 2018, Dartmouth-Hitchcock Health had 39 participating specialties. Psychiatric eConsult procedures and protocols were developed based on established guidelines set forth by the AAMC and used by all specialties within Dartmouth-Hitchcock Health. These included referral template guidelines, quality metrics for response content and time frames, and the expectations of referring providers and responding specialists. eConsult templates were created for questions regarding depression, anxiety, substance use, attention-deficit/hyperactivity disorder, bipolar disorder, and psychosis. Templates include a free-text field for the specific clinical question and short checklists for common comorbid conditions and prior medication trials. Patient-reported measures such as the Patient Health Questionnaire 9 and General Anxiety Disorder 7 are commonly available in the EHR, and relevant laboratory data are automatically pulled by the EHR into the referral template. eConsults are submitted to a shared pool within the EHR, which is monitored daily by two psychiatrists. The responding psychiatrist reviews the specific consultation question and information in the eConsult referral template and may also choose to review additional clinical information available in the EHR, such as the PCP’s most recent progress note. An example eConsult is shown in Textbox 1, with a standard disclaimer included at the end of every eConsult in Textbox 2. Response time is expected within 3 business days (72 hours) and both the request and response become part of the patient’s EHR. Typically, the specialist response to the PCP completes the eConsult exchange. Further clinical management is conducted by the PCP. However, in a small minority of cases, the consulting psychiatrist may recommend an additional referral for in-person consultation. Dartmouth-Hitchcock Health provides an internal accounting of a nominal work relative value unit credit for both the PCP and consulting psychiatrist, based on the time taken to complete the e-consultation. e-Consultations serve as no additional cost to the patient, and the insurance company of the patient is not billed.

**Example of a psychiatric eConsult.**

1. eConsult question(s): Patient currently takes citalopram 40 mg daily. She has been on this dose since June of 2012. She shares that it may not be as effective for her mood as it used to be. She suffers from chronic neck pain with radiculopathy. She desires to switch to duloxetine, with the hope of gaining better pain control. Can you please advise on a cross taper from 40 mg citalopram to duloxetine?

2. Recommendation(s): Cross tapering an anti-depressant is often an art more than a science. I usually take into account a patient's sensitivity to medication side effects when doing a cross taper.

Assuming that this patient is not particularly sensitive to medication side effects, I would suggest the following:

Week 1: citalopram 20mg po daily and duloxetine 20mg po daily

Week 2: citalopram 10mg po daily and duloxetine 40mg po daily

Week 3: Discontinue citalopram and increase to duloxetine 60mg po daily

You can speed up or slow down this taper depending on how the patient tends to respond to these sorts of medication changes. I would slow down the taper if she is having any side effects. I would also educate about the risk for serotonin syndrome, as a cross taper like this puts the patient at a slightly increased risk for serotonin syndrome compared to tapering and discontinuing the citalopram first and then starting duloxetine.

**Standard disclaimer enclosed at the end of each eConsult.**

This eConsult is focused on the specific clinical question(s) asked by the referring clinician, is based on the clinical data available to me, the consulting physician, at the time of the request, and is furnished without benefit of a comprehensive evaluation or physical examination of the patient by me. The guidance set forth in the eConsult note will need to be interpreted in light of any clinical issues not known to me or any changes in patient status that I may not be aware of at the time of filing this eConsult. If further consultation is necessary, an in-person visit with me or another member of our group is an option.

### Data Collection Plan

We performed content analyses of all eConsults to psychiatry that occurred between May 2016 and February 2019 at Dartmouth-Hitchcock Medical Center (DHMC), located in Lebanon, New Hampshire. We included all eConsults for patients18 years or older, totaling 343 eConsults.

We reviewed and categorized eConsults according to the following: consult diagnosis, question type, recommendations, outcome classification, implementation status, time frame, and distance.

- “Consult diagnosis” was the listed billable diagnosis placed by the PCP at the time of the consult. This diagnosis did not always meet the Diagnostic and Statistical Manual of Mental Disorders (Fifth Edition; DSM-5) criteria, given the limited information in the medical chart required to fully meet criteria for specific diagnoses.
- “Question type” assessed the specific type of question asked by the PCP. This aspect of the eConsult was further categorized to distinguish and group the various types of questions. All eConsult “question types” were independently assessed by two raters and categorized as confirming or making a diagnosis, medication recommendation, medication side effects or interactions, referral for psychotherapy, navigation of the health care system, need for psychiatric in-person evaluation, or other. Navigation of the health care system question types were categorized as all questions where a PCP requested assistance from the eConsult psychiatrist to determine the appropriate level of care or specific services that a patient might require based on their presentation. Medication recommendation was further categorized as pharmacological recommendation, assistance with a medication taper, or validation of current medication treatment plan.
- “Recommendations” outlined the specific recommendation type made by a psychiatrist to the PCP. These were categorized as one or more of the following: psychotherapy, medication choice, need for diagnostic clarity or additional testing, face-to-face consultation, further workup needed, no changes to plan, or other.
- “Implementation status” categorized whether or not the primary care clinician implemented the recommendations. All records were reviewed to evaluate the consulting psychiatrist recommendation and whether that specific recommendation was implemented by the PCP. If the PCP documented within the EHR that they followed the specific recommendation by the psychiatrist (eg, ordered recommended medication, referred to recommended provider, or gathered additional information for diagnostic clarification), they were documented to have “implemented recommendation.” If the recommendations made by the psychiatrist were not implemented by the PCP, the reason for lack of implementation was explored, and they were documented to “have not implemented the recommendation.” If there was no information in the EHR as to whether or not a recommendation was implemented either due to a loss of follow-up or lack of documentation in the chart by the PCP, the eConsult was excluded from this category.
- “Time frame” calculated the time it took from placement of the eConsult until completion of the eConsult by the consulting psychiatrist. We documented the number of exchanges between a consulting psychiatrist and primary care physician. A standard exchange was documented as zero if the only exchange was the initial consult question and a response back to the consult question.
- “Distance” was the distance in miles between the PCP office from whence the eConsult originated and DHMC, where the eConsult psychiatrist was located. The distance between the patient’s hometown and the location of the eConsult psychiatrist was also calculated.

### Data Analysis

All information from charts were obtained via chart abstraction. We had three independent reviews for all consultations (two medical students and one attending psychiatrist). We used descriptive statistics to report patient demographics, consult diagnosis, question type, psychiatrist recommendation, recommendation implementation, time frame, and distance of primary care physician and patient from consulting provider.

## Results

### Patient Characteristics

Patient age ranged from 18 to 97 years. Most of the 343 patients were female (n=241, 70.3%), were non-Hispanic White (n=325, 94.8%), were employed full-time (n=157, 45.8%), were married (n=114, 42%), and had commercial insurance (n=212, 61.8%). All patients preferred English (Table 1).

**Table 1:** Patient characteristics of psychiatry eConsults from 2016 to 2019.

Characteristics			Patients (N=343)
Age (years), median (range)			47 (18-97)
**Gender, n (%)**			
	Female	241 (70.3)	
	Male	100 (29.2)	
	Female (transgender)	1 (0.3)	
	Male (transgender)	1 (0.3)	
**Race, n (%)**			
	White (non-Hispanic)	325 (94.8)	
	White (Hispanic)	4 (1.2)	
	Asian	5 (1.5)	
	African American	4 (1.2)	
	Other	5 (1.5)	
**Employment status, n (%)**			
	Employed (full-time)	157 (45.8)	
	Employed (part-time)	15 (4.4)	
	Not employed	71 (20.7)	
	Retired	70 (20.4)	
	Disabled	12 (3.5)	
	Other (not listed)	18 (5.2)	
**Insurance, n (%)**			
	Commercial insurance	212 (61.8)	
	Dual eligibility (Medicare and Medicaid)	7 (2.0)	
	Medicaid	31 (9.0)	
	Medicare	78 (22.7)	
	No insurance	15 (4.4)	
**Marital status, n (%)**			
	Divorced	45 (13.1)	
	Married	144 (42.0)	
	Other	23 (6.7)	
	Separated	7 (2.0)	
	Single	124 (36.2)	
**Preferred language, n (%)**			
	English	340 (99.1)	
	Other	3 (0.9)	

### eConsult Content and Characteristics

#### Consult Diagnosis

eConsults often included multiple diagnoses. Therefore, among 343 eConsults, 450 diagnoses were abstracted. The most common diagnosis was depression (n=162, 36%), followed by anxiety (n=118, 26.2%), psychosis (n=47, 10.4%), other (n=47, 10.4%), bipolar disorder (n=28, 6.2%), attention-deficit/hyperactivity disorder (n=24, 5.3%), substance use (n=12, 2.7%), and posttraumatic stress disorder (n=12, 2.7%). “Other” diagnoses included eating disorders (n=11), personality disorders (n=5), obsessive-compulsive disorder (n=5), neurocognitive disease (n=8), insomnia (n=7), unspecified mood disorder (n=9), and adverse drug reactions (n=2). DSM-5–specific diagnoses were often not used, as there often was not sufficient information in the medical chart to make a DSM-5 diagnosis.

#### Question Type

A majority (n=288, 84%) of the 343 eConsults pertained specifically to medication management, which included medication choice, side effects or interactions, or medication taper. eConsults seeking help in securing a psychiatry referral represented 6.4% (n=22) of eConsults, and eConsults dealing with navigating the health care system, including questions on appropriate referrals or available services, represented 4.6% (n=16) of eConsults. Only 1.7% (n=6) of eConsults asked for assistance in making a clinical diagnosis, and 1.2% (n=4) were a request for referral for psychotherapy. Finally, 2% (n=7) of eConsult questions were classified as “other” and included management strategies (eg, how to approach a patient in a clinic who does not recognize their own delusions) and verification of a management plan.

#### eConsult Recommendations

Each eConsult response typically included multiple recommendations, with a total of 602 recommendations collected across 343 eConsults. Most eConsults recommended medication management, psychotherapy, or a combination of both (419/602, 69.6%). The consulting psychiatrist recommended to either reassess the diagnosis or obtain additional history 15% (90/602) of the time; face-to-face consultations or referrals to outpatient therapy were recommended 7% (43/602) of the time. No changes were recommended in 1.7% (10/602) of the eConsult responses. The category “other” included referral to specialized resources (eg, aging resource center), direct phone call to PCP, and other treatment modalities (eg, light box), and was recommended 1.7% (10/602) of times.

#### eConsult Implementation Status

A total of 43 out of 343 of eConsult cases could not be evaluated for implementation status due to a loss of follow-up or lack of documentation in the chart by the PCP. At least one of the psychiatrist’s recommendations was implemented by the PCP in 282 out of 300 of cases. The recommendation was specifically not implemented in 6% (18/300) of the cases due to patient refusal (n=14), PCP refusal (n=2), and the patient improving without intervention (n=2). Therefore, of the 300 eConsults available for analysis, at least one recommendation was implemented 94% (282/300) of the time.

#### eConsult Time Frame

The average time for a psychiatrist to respond to an eConsult was 25.8 (IQR 5.4-28.5) hours. Nearly all eConsults (330/343, 96.2%) were sufficiently answered with one response.

#### Distance of Primary Care Physician and Patient From Consulting Provider

The average distance of the referring PCP office was 46 miles away from the psychiatrist who answered the eConsult. Of the 330 patients with listed PCPs, 41% (n=142) of eConsults were from PCPs with offices in the same hospital as the answering psychiatrist, and 35.8% (n=118) of eConsults were placed by PCPs in offices greater than 50 miles away. The average distance of the patient’s hometown from the answering psychiatrist was also analyzed to measure the distance a patient would have to travel to attend an in-person evaluation by one of our psychiatrists. A total of 8 patients were excluded from this analysis due to lack of hometown data, resulting in an average distance of 43.7 miles from patient hometown to consulting psychiatrist. We found that 29% (97/335) of patients lived within 15 miles of the consulting psychiatrist, and 42% (142/335) lived more than 50 miles away.

## Discussion

Our study demonstrates that psychiatric e-consultation can be a helpful tool to support PCPs in delivering mental health care to their patients. Psychiatric e-consultation serves as a bridge to improve timely access to specialist opinion, aiding both PCPs and patients. The asynchronous nature of eConsults permits the PCP to ask and the consulting psychiatrist to answer eConsults at a time convenient to both providers, rather than having to respond to a page or phone call from the PCP who is requesting the consultation. At our institution, the eConsult psychiatrists are outpatient psychiatrists who are not embedded in primary care practices and are often not available to discuss a case with a PCP by phone. Though eConsults do not replace the benefits of having a psychiatrist embedded in a primary care practice and available to do curbside consultations, they offer access to psychiatric care for primary care clinicians who do not have easy access to a psychiatrist.

e-Consultation may be of particular benefit in rural settings, where access to psychiatric specialists is often limited. Our analysis shows that eConsults can be answered promptly (on average <26 hours), and the vast majority of recommendations (94%) are implemented by primary care physicians. Considering in-person or telehealth psychiatry referrals for initial evaluations can take months to schedule, the timeliness of e-consultation can be helpful in reducing delays initiating treatment for common mental health disorders that frequently present in primary care. With additional constraints on in-person care caused by the COVID-19 pandemic, e-consultation provides yet another tool that may be used to meet the growing mental health needs in the United States.

Over 80% of eConsults in our study were questions about the management of psychiatric medications, which is similar to the findings of other studies evaluating the content of psychiatry eConsults [8]. Specifically, most PCPs in our study submitted an eConsult for medication recommendation or a second opinion on their choice of medication for an established diagnosis. Less commonly, questions were asked about side effect profile or advice on tapering a certain medication. Our finding that most eConsult questions were regarding pharmacotherapy may be explained by the therapeutic complexity of managing patients with psychiatric conditions.

Our response time was similar to those of other studies evaluating psychiatry eConsults, with an average response time of 1.1 days compared to 1.4 days in a study by Wren et al [9] and 2.6 days in a study by Lowenstein et al [10]. Furthermore, our response time to eConsults may have been slightly increased due to eConsults placed outside of normal work hours where response was delayed until the next workday. For reference, eConsults were sometimes returned as early as 12 minutes, and 31.2% (107/343) were returned within 12 hours. Overall, the quick turnaround time both in our study and in the literature confirms the viability of eConsults as a means to get more timely psychiatric recommendations than an in-person evaluation.

We believe that our study’s 94% implementation rate of at least one eConsult recommendation and the fact that 96% of eConsults did not prompt further questions or follow-up from the PCP demonstrates that the quality of recommendations still remain high despite the lack of in-person evaluation, which is congruent to the 96% implementation rate reported by Lowenstein et al [10]. However, satisfaction of our eConsults would be better evaluated with a survey to PCPs, which was not performed. A follow-up study should be conducted to measure PCP satisfaction and patient outcomes following implementation of eConsult recommendations.

The utility of psychiatric eConsults particularly in rural settings is substantial. In our study, both patients and PCP physician offices were on average nearly an hour away from the center where the consulting psychiatry providers were located. Therefore, providing PCPs with the tool to receive timely specialist advice and potentially obviate the need for in-person evaluation has the potential to provide more immediate access to mental health care, reduce additional costs or need for separate outpatient appointments, and potentially reduce additional costs either for transit or time off from work.

Limitations to this study include the fact that this is a single-center study in a rural academic medical center, which is susceptible to a lack of external generalizability when compared to medical centers in other settings. eConsults were also performed by two psychiatrists, so certain elements of the consult such as the time to respond may differ if more consulting psychiatrists were included. Finally, this was a retrospective study evaluating only patients 18 years or older and from a 3-year period.

Psychiatric e-consultations are effective in delivering specialist recommendations to PCPs and patients in a timely manner. eConsults may be of particular benefit in rural settings, bridging both time and distance, which are common barriers that delay care. eConsults allow PCPs to quickly verify or make changes to their management of psychiatric disorders and may avoid the need for in-person appointments with a psychiatric specialist in a majority of cases. Demonstrating the value and effectiveness of psychiatric e-consultation is especially important given the barriers patients and PCPs may experience when trying to refer for in-person psychiatry evaluation.

## Abbreviations

AAMC: Association of American Medical Colleges
CORE: Coordinating Optimal Referral Experiences: Implementing eConsults and Enhanced Referrals
DHMC: Dartmouth-Hitchcock Medical Center
DSM-5: Diagnostic and Statistical Manual of Mental Disorders (Fifth Edition)
EHR: electronic health record
PCP: primary care provider
